# Safety and preliminary efficacy data of a novel Casein Kinase 2 (CK2) peptide inhibitor administered intralesionally at four dose levels in patients with cervical malignancies

**DOI:** 10.1186/1471-2407-9-146

**Published:** 2009-05-13

**Authors:** Ana M Solares, Agueda Santana, Idania Baladrón, Carmen Valenzuela, Carlos A González, Alina Díaz, Dagnelia Castillo, Thelvia Ramos, Roberto Gómez, Daniel F Alonso, Luis Herrera, Hugo Sigman, Silvio E Perea, Boris E Acevedo, Pedro López-Saura

**Affiliations:** 1 Gyneco-obstetric Hospital Clodomira Acosta Ferrales (National Reference Center for Cervical Cancer Research), Havana, Cuba; 2 Gyneco-obstetric Hospital 10 de Octubre, Havana, Cuba; 3 Center for Biological Research, Clinical Trials Division, Havana, Cuba; 4 National Center for Toxicology, Havana, Cuba; 5 Gyneco-obstetric Hospital Ramón González Coro, Havana, Cuba; 6 ELEA Laboratories, Buenos Aires, Argentina; 7 National University of Quilmes, Buenos Aires, Argentina; 8 Center for Genetic Engineering and Biotechnology, Havana, Cuba

## Abstract

**Background:**

Cervical cancer is now considered the second leading cause of death among women worldwide, and its incidence has reached alarming levels, especially in developing countries. Similarly, high grade squamous intraepithelial lesion (HSIL), the precursor stage for cervical cancer, represents a growing health problem among younger women as the HSIL management regimes that have been developed are not fully effective. From the etiological point of view, the presence of Human Papillomavirus (HPV) has been demonstrated to play a crucial role for developing cervical malignancies, and viral DNA has been detected in 99.7% of cervical tumors at the later stages. CIGB-300 is a novel cyclic synthetic peptide that induces apoptosis in malignant cells and elicits antitumor activity in cancer animal models. CIGB-300 impairs the Casein Kinase (CK2) phosphorylation, by targeting the substrate's phosphoaceptor domain. Based on the perspectives of CIGB-300 to treat cancer, this "first-in-human" study investigated its safety and tolerability in patients with cervical malignancies.

**Methods:**

Thirty-one women with colposcopically and histologically diagnosed microinvasive or pre-invasive cervical cancer were enrolled in a dose escalating study. CIGB-300 was administered sequentially at 14, 70, 245 and 490 mg by intralesional injections during 5 consecutive days to groups of 7 – 10 patients. Toxicity was monitored daily until fifteen days after the end of treatment, when patients underwent conization. Digital colposcopy, histology, and HPV status were also evaluated.

**Results:**

No maximum-tolerated dose or dose-limiting toxicity was achieved. The most frequent local events were pain, bleeding, hematoma and erythema at the injection site. The systemic adverse events were rash, facial edema, itching, hot flashes, and localized cramps. 75% of the patients experienced a significant lesion reduction at colposcopy and 19% exhibited full histological regression. HPV DNA was negative in 48% of the previously positive patients. Long term follow-up did not reveal recurrences or adverse events.

**Conclusion:**

CIGB 300 was safe and well tolerated. This is the first clinical trial where a drug has been used to target the CK2 phosphoaceptor domain providing an early proof-of-principle of a possible clinical benefit.

## Background

Current trends in cancer treatment focus to "targeted therapy", referring to a new generation of drugs designed to interfere with specific molecular targets that are necessary to maintain malignant phenotype, progression or metastasis [1]. Protein kinases constitute promising classes of cancer targets as some specific inhibitors have shown clinical benefit in patients [2,3]. CK2 is involved in the tumor cell adaptive response and protection against apoptosis [4]. It is uniformly deregulated 3- to 7-fold in different cancer types [5] and has been associated with aggressive tumor behavior in human head and neck carcinoma [6]. E7 viral oncoprotein phosphorylation by CK2 plays an important role on HPV malignant properties [7]. Although no previous clinical trials with CK2 inhibitors have been reported so far, promising preclinical results indicate that it could be a good molecular target for cancer therapy [8].

CIGB-300 is a cyclic peptide that impairs CK2 phosphorylation after intracellular delivery. It behaves as pro-apoptotic both on HPV-positive and -negative tumor cell lines. Intralesional injections of the drug induced a vigorous antitumor effect on HPV-positive solid tumors in mice [9]. Therefore, it was hypothesized that local CIGB-300 injections could exhibit clinical benefit in patients with cervical malignancies.

Being the first-in-human application of CIGB 300 or any CK2 inhibitor, the trial reported here was done to investigate the safety and tolerability of this peptide-based drug after local injections. Besides, efficacy signs were also explored in order to provide an early proof-of-principle for CIGB-300 as a modality of targeted cancer therapy in cervical malignancies.

## Methods

The study followed the principles of the Declaration of Helsinki for investigations in humans. It was approved by the Ethics and Scientific Committees of the participant institutions and by the Cuban Regulatory Authority.

Patients with colposcopical and cytological suspect of HSIL or microinvasive cervical cancer were recruited at specialized services from seven gyneco-obstetric hospitals throughout Havana and other provinces. They were then evaluated at the National Reference Center for Cervical Cancer at the "Clodomira Acosta" Hospital, where the eligibility criteria were verified. Product administrations and evaluations took place, as in-patients, at the National Center for Toxicology, in a specialized facility for clinical Pharmacology studies.

### Patient Eligibility

Patients were included if they were 18 to 65 years-old, had histological diagnosis of HSIL or epidermoid microinvasive cervical cancer (FIGO Classification Stage 1A1)[10], with larger diameter ≥ 3 mm by video-colposcopy, corresponding with major changes or infiltrating stage according to the International Federation of Cervical Pathology and Colposcopy [11], and gave their written, informed consent to participate. Exclusion criteria were to have received chemotherapy or surgical, ablative, radiant, or immunomodulator treatment, up to 3 months before inclusion, psychiatric dysfunctions, pregnancy and breast-feeding, chronic diseases such as asthma, epilepsy, autoimmune diseases, hypertension, anemia, acute systemic or genital tract infections, renal, hepatic and cardiovascular disorders

### Study Design and Treatment Plan

The design was an open, uncontrolled, dose-escalating trial. The four dose levels (14, 70, 245 and 490 mg) were tested sequentially. The protocol previewed that the next dose level would be undertaken after the safety of the previous one was assured when no severe adverse events (AE) occurred up to 20 days after the first administration. Sample size for each group was previewed as between 7 and 10 patients. This N range assured that if no severe adverse reaction appeared, the probability of its occurrence would be less than 20%, with an 80%–90% confidence interval. Since patients of the same group were treated the same days, the exact number of subjects in each group was finally determined by practicalities such as the number of patients recruited that week.

CIGB-300 was synthesized and supplied by the Center for Genetic Engineering and Biotechnology in 245 mg vials, as a lyophilized powder. It was reconstituted with 1 mL of water for injection and subsequent dilutions were prepared to a final volume of 1 mL for groups I and II that received less than 245 mg per dose. Groups III and IV received one and two vials per administration, respectively.

One milliliter containing the corresponding drug dose was evenly distributed among four previously defined quadrants on the cervical lesion area. Injections were approximately 2 mm deep and were given for 5 consecutive days. Fifteen days after treatment cessation all patients underwent conization by loop electrosurgical excision procedure (LEEP). Concomitant interventions could be taken to manage the AE, according to established clinical practices. Since allergic-like reactions appeared with the lower dose levels, prophylactic antihistaminic medication (50 mg intramuscular diphenhydramine) was given 20 minutes before CIGB 300 injections to groups III and IV.

### Evaluation

Pretreatment evaluation included a detailed history, physical and colposcopical examinations, and HPV testing. Besides, electrocardiogram, hematological counts, blood chemistry, coagulation and cervix microbiological studies were done. Twenty-four hours after the fifth CIGB-300 administration and 15 days after treatment cessation these evaluations were repeated. Additionally, patients were followed clinically and colposcopically at 3, 6, 9, and 12 months after the conization. Papanicolau's test was done at these moments as well.

Since the main purpose of the trial was to evaluate the product's safety, local and systemic AE were carefully screened up. A colposcopic examination of the whole cervix area was carefully performed daily during the consecutive 5-day drug regimen, 24 hours after the last administration and 15 days later. Likewise, systemic toxicity was evaluated during 24 hours after each CIGB-300 administration, including cardiovascular monitoring during the injections and vital signs measurements (temperature, heart and respiratory frequencies, and blood pressure) 30 min. after the end of the injection, then every hour during 4 hours and every 4 hours thereafter.

The medical terminology for AE and their severity classification (in grades) used were those of the Cancer Therapy Evaluation Program, Common Terminology criteria [12]. In general, this classification consists in 5 grades: (1) mild if no therapy is necessary, (2) moderate if specific treatment is needed, (3) severe when hospitalization or its prolongation was required, (4) very severe if a reaction was potentially life-threatening, and (5) when contributed to the patient's death.

A qualitative assessment was used to classify the causal relationship as definite, probable, possible or doubtful [13]. A reaction was classified as "definite" if (1) followed a reasonable temporal sequence after drug administration; (2) followed a known pattern of response to the drug; (3) could not be explained by concurrent disease or other drugs; and (4) was confirmed by improvement upon drug removal and by reappearance on re-challenge. It was considered as "probable" if it had criteria (1), (2), (3) and was confirmed on drug suspension but not on re-challenge. A reaction was defined as "possible" if followed a reasonable time sequence to the drug administration, but could also be explained by concurrent disease or other drugs. Finally, a reaction that was more likely related to factors other than the suspected drug was classified as doubtful.

Colposcopical evaluations were done using a video colposcopy device (Mediscope, Medison, Korea) with a calibrated rack to standardize imaging distance and illumination. Lesions were examined by two different specialists after applying 5% acetic acid for 3 min. They were described according to the Barcelona 2002 classification [11] and their morphometric analysis was done with validated software for quantitative digital image evaluation (MADIP^® ^V.4; Institute of Cybernetics, Mathematics and Physics, Havana)

Histological analyses were performed on colposcopically directed biopsies from abnormal areas taken before treatment and afterwards in the cone specimen. Permanent sections were subsequently prepared, stained with hematoxylin-eosin and finally reviewed by two different pathologists who classified the lesions according to the FIGO Classification.

The presence of HPV DNA in biopsies was examined before and after CIGB-300 treatment by PCR using a pair of L1-consensus primers (GP5+/GP6+) [14]. The HPV DNA detection limit was about 310 viral copies. Additional PCR with specific HPV-16 and -18 primers was performed for typing [15] and HPV DNA levels were normalized with those corresponding to β-globin as housekeeping gene.

### Response Criteria and Clinical Activity

The total lesion area (TLA) was the parameter evaluated for colposcopical response. Response was defined according to the RECIST criteria [16]. Complete response was considered as disappearance of all high grade lesions; partial response if at least a 30% TLA decrease was observed; stable disease if changes were within -30% and + 20% regarding initial TLA; progressive disease meant at least a 20% TLA increase.

The histological response was based on the lesions phase changes. A complete response was defined as the absence of high grade lesions 15 days after drug administration; partial response when a down-staging of the high grade lesion occurred, and progression if there was up-staging.

### Statistics

It was not an objective of the study to compare the effect of the different CIGB-300 doses among them but to test each one in terms of tolerability and a preliminary efficacy evaluation. Therefore, dose groups were sequential and not the product of randomization.

Continuous variables were analyzed for normal distribution using the Shapiro-Wilk's test. They are presented as mean ± standard deviation (SD) or median ± interquartile range (QR). Categorical variables are presented as proportions. The correlation between the number of local AE and the total dose was estimated using the Pearson's correlation coefficient. The level of significance chosen was 0.05.

## Results

### Patient Characteristics and Dosing History

Eighty patients from the seven participating recruiting centers were evaluated from January to July 2006. Of them, 31 were enrolled. Their demographic and baseline characteristics are outlined in Table 1. Age ranged from 20 to 54 years and lesion area between 18 and 319 mm^2^. Most of the lesions (16; 52%) were carcinoma *in situ*; 14 (45%) were CIN and there was one microinvasive carcinoma. HPV DNA was detected in 22/28 biopsies (79%; 3 samples were not useful for PCR). All were HPV 16 except for one HPV 18 and two non 16/non 18 types.

**Table 1:** Individual patient data

**Patient No**.	CIGB-300 (mg)	Age	Total lesion area (mm^**2**^)		Histological diagnosis		HPV status	
			
			Initial	End	Initial	End	Initial	End
1	14	28	289.1	187.1	CIN III	CIN III	16	16
2	14	42	186.9	117	CIN III	MIC	16	16
3	14	35	165.3	126.05	CIN III	CIN III	Neg	Neg
4	14	33	128.6	69.59	CIN III	CIN III	16	16
5	14	33	17.7	13.4	MIC	CIN III	Neg	Neg
6	14	31	103.3	0	CIN III	Koilocytosis	16	Neg
7	14	43	93.2	45.8	CIN III	CIN III	16	16
8	14	40	62.3	17.1	CIN III	CIN III	16	Neg
9	70	34	64.8	19.6	CIN III	CIN III	16	Neg
10	70	52	72.8	5.1	CIN II	Koilocytosis	16	16
11	70	30	55.9	21.5	CIN II	CIN II	Neg	Neg
12	70	54	36.8	7.6	CIN II	CIN II	Neg	Neg
13	70	36	39.6	0	CIN II	CIN I	16	Neg
14	70	20	36.1	0	CIN III	Koilocytosis	Neg	16
15	70	27	73.3	38.4	CIN III	CIN III	16	Neg
16	70	27	93.6	34.1	CIN III	CIN III	N16-N18	Neg
17	245	31	219	134	CIN III	CIN III	16	16
18	245	40	80.7	5	CIN III	CIN III	16	16
19	245	33	87.8	77	CIN III	CIN III	16	Neg
20	245	26	176	8.11	CIN III	CIN III	N.D.	N.D.
21	245	40	85	0	CIN III	CIN III	18	18
22	245	21	30.2	0	CIN III	Neg	N.D.	N.D.
23	245	26	64.8	0	CIN III	Koilocytosis	N.D.	N.D.
24	490	41	247	38	CIN II	CIN III	Neg	Neg
25	490	29	319	77	CIN II	CIN III	16	Neg
26	490	31	213	38	CIN II	MIC	16	Neg
27	490	33	23	5	CIN III	CIN II	16	16
28	490	39	215	73	CIN III	CIN III	16	N.D.
29	490	46	30.3	2.1	CIN III	CIN III	N16-N18	Neg
30	490	40	249	55	CIN III	CIN III	16	16
31	490	42	144	38	CIN III	CIN III	16	16

Abbreviations: CIN II-III (cervical intraepithelial neoplasia, grade II or III), MIC (microinvasive carcinoma), Neg: Negative, N16-N18: Non-16 Non 18, ND (not determined)

The treatment schedule was completed in 30 patients (97%). One did not conclude the assigned scheme (490 mg per dose, group IV), due to a transient vasovagal syndrome with loss of consciousness that was classified as a severe event (grade 3). She received only 77 mg of the drug at the first application and the interruption was permanent.

### Local and Systemic Toxicity

All patients experimented some local or systemic AE. A total of 409 events were recorded, 337 (82%) mild, 71 (17%) moderate and only one AE was classified as severe.

The most frequent local AE were bleeding, pain on lower abdomen, hematoma, ulcer, necrosis, erythema, and edema on the injection site (Table 2). There was a significant correlation with respect to the total dose received and the number of local AE (r^2 ^= 0.469, *p *= 0.008). All local AE were grade 1 except for 2 ulcers in group II, 2 ulcers, 2 hematoma and 2 necrosis in group III, and 2 hematoma and necrosis in group IV, which reached grade 2. Twenty-two patients had either local or systemic grade 2 AE. The colposcopic examination 15 days after CIGB-300 treatment evidenced a complete recovery of all the local AE.

**Table 2:** Frequency and distribution of the local adverse events (percents refer to the total number of events within each group)

Local Events	Group I (14 mg)	Group II (70 mg)	Group III (245 mg)	Group IV (490 mg)
Bleeding	9 (47.4%)	22 (57.9%)	5 (14.7%)	16 (39.0%)
Pain in lower abdomen	0	6 (15.8%)	10 (29.4%)	8 (19.5%)
Hematoma	0	3 (7.9%)	7 (20.6%); g2: 1 (2.9%)	4 (9.8%); g2: 1 (2.4%)
				
Ulcer or superficial necrosis	0	6 (15.8%); g2: 6 (15.8%)	3 (8.8%); g2: 3 (8.8%)	6 (14.6%); g2: 4 (9.8%)
Erythema in situ*	2 (10.5%)	0	3 (8.8%)	6 (14.6%)
Edema in situ*	4 (21.0%)	0	3 (8.8%)	1 (2.4%)
Vaginal burning	2 (10.5%)	0	2 (5.9%)	0
Spontaneous peeling	1 (5.3%)	1 (2.6%); g2: 1 (2.6%)	0	0
				
Pain in situ*	1 (5.3%)	0	0	0
Color change at puncture site	0	0	1 (2.9%)	0

**Total**	**19**	**38**	**34**	**41**

*: *In situ *events referrer to the localization of such events at the site of injection. All events are grade 1 (mild) if not specified; g2: grade 2 (moderate).

Table 3 lists the clinically relevant systemic toxicities observed during the study. The most frequent were allergic-like reactions including rash, facial edema, itching, hot flashes, extensive bumps, localized cramps and tachycardia. The rate of these AE decreased with the subsequent CIBG 300 administrations (data not shown). Prophylactic antihistaminic medication greatly ameliorated some of the systemic allergic-like AE in cohorts III and IV (localized rash and localized bumps) (Table 3). Four vasovagal syndromes were observed; one of them was grade 3 since the patient lost her consciousness transiently (1 min). Its association to CIGB-300 was not demonstrated.

**Table 3:** Frequency and distribution of the systemic events occurred at each dose level

Systemic Events	Group I (14 mg)	Group II (70 mg)	Group III (245 mg)	Group IV (490 mg)	
Itching	8 (14.6%)	18 (25.0%)	29 (25.9%)	29 (32.3%)	84
Hot flashes	12 (21.8%)	14 (11.7%)	8 (7.1%)	8 (8.9%)	42
Localized rash	3 (5.4%)	21 (17.5%)	6 (5.4%)	8 (8.9%)	38
Localized facial edema	9 (16.4%)	6 (5.0%)	8 (7.1%)	13 (14.4%)	36
Bumps	0	12 (10.0%)	17 (15.2%)	6 (6.7%)	35
Extensive rash	2 (3.6%)	0	21 (18.8%)	5 (5.6%)	28
Cramps	3 (5.4%)	2 (1.7%)	11 (9.0%)	6 (6.7%)	22
Headache	3 (5.4%)	2 (1.7%)	4 (3.6%)	2 (2.2%)	11
Metallic flavor	4 (7.3%)	6 (5.0%)	1 (0.9%)	0	11
Tickling or tingling	5 (9.1%)	6 (5.0%)	0	0	11
Tachycardia	0	7 (5.8%)	3 (2.7%)	0	10
Tinnitus	0	7 (5.8%)	0	0	7
Nausea	0	3 (2.5%)	1 (0.9%)	3 (3.3%)	7
Facial erythema	0	7 (5.8%)	0	0	7
Palpitations	0	2 (1.7%)	1 (0.9%)	2 (2.2%)	5
Hypertension	3 (5.4%)	1 (0.8%)	0	1 (1.1%)	5
Vasovagal syndrome	0	g2: 2 (1.7%)		g3: 2 (2.2%)	4
Dizziness	1 (1.8%)	2 (1.7%)	0	0	3
Tremors	0	0	0	3 (3.3%)	3
Vomiting	0	2 (1.7%)	0	0	2
Fast breathing	1 (1.8%)	0	0	0	1
Abdominal pain	1 (1.8%)	0	0	0	1
Arrhythmia	0	0	0	1 (1.1%)	1
Hypotension	0	0	1 (0.9%)	0	1
Posture-related tremor in lower limbs	0	0	0	1 (1.1%)	1
Red eyes	0	0	1 (0.9%)	0	1

Total	55	120	112	90	

There were no differences between baseline and post-treatment blood counts, serum chemistry results, coagulation parameters and electrocardiograms (data not shown).

### Efficacy analysis

Individual efficacy results are shown in Table 1 and summarized in Table 4. TLA decreased in all patients at colposcopy 15 days after CIGB-300 treatment. Colposcopic response was evidenced in 28/31 patients (90%), six of them (19.4%) complete. Response rates were above 75% in all groups. Histological analyses indicated that 19.3% of the patients (6/31) experienced full regression after treatment while 25.8% (8/31) had histological grade down-staging. Responses were 10/16 (62.5%) for cases with carcinoma and 5/9 (55.6%) for patients with CIN. HPV status became undetectable in 48% (10/21) of the initially HPV-positive patients from all dose cohorts. One patient was not evaluated after treatment.

**Table 4:** Efficacy evaluation of CIGB-300 administration.

		Group I (14 mg)	Group II (70 mg)	Group III (245 mg)	Group IV (490 mg)
TLA (mean ± SD)	Initial (mm^2^)	15.7 ± 10.4	7.6 ± 2.7	10.0 ± 7.8	14.5 ± 15.5
	Final (mm^2^)	9.9 ± 19.6	2.8 ± 3.9	1.4 ± 14.0	5.0 ± 5.9
	% Reduction	41.6 ± 41.3	74.6 ± 36.2	95.4 ± 61.2	81.3 ± 19.7

Responders	Colposcopy	6/8 (75.0%)	8/8 (100%)	6/7 (85.7%)	8/8 (100%)
	Histology	1/8 (12.5%)	3/8 (37.5%)	2/7 (28.6%)	0/8 (0.0%)
	HPV status	2/6 (33.3%)	4/5 (80.0%)	1/4 (25.0%)	3/6 (50.0%)

### Long term follow-up

None of the patients showed confirmed lesion recurrence during the 12-months follow-up. There were no late adverse events either. Interestingly, 4 patients became pregnant, two of them with previous infertility history, with successful, normal neonates.

## Discussion

CIGB-300, a novel inhibitor of the CK2 phosphorylation, was safe and well tolerated in this study. No severe drug-related adverse reactions were observed. Intralesional application of CIGB-300 at a daily dose range from 14 to 490 mg through a consecutive-5 day regimen seems to be a safe approach. The dose range and therapeutic regimen were selected according to an extrapolation made from previous toxicological (data not published) and pharmacodynamic studies in animal models. It was not an objective of the study to compare the effect of the different CIGB-300 doses among them but to test each one in terms of tolerability and a preliminary efficacy evaluation. Although neither the MTD nor DLT were found, the most frequent systemic and local toxicities produced by the intralesional administration of CIGB-300 were identified,

The appearance of some transient systemic "allergic-like" events could be explained by a potential histamine release from local mastocytes. Accordingly, prophylactic antihistaminic medication of patients exposed to the higher dose levels ameliorated the systemic localized rash and bumps. As CIGB-300 inhibits CK2 phosphorylation, inducing apoptosis on the target cells, mastocyte death and the subsequent histamine release to the circulation should not be discarded and merits further investigation. However, other drugs such as interferons [17-19], which induce apoptosis by other mechanisms that exclude CK2, have been also locally administrated on the cervix without systemic "allergic-like" reactions. Alternatively, this syndrome could be related to CIGB 300's molecular structure, composed by several predominantly positively charged aminoacids that make it a very basic peptide and, therefore, behave as a potential histamine releaser [20].

The vasovagal events (n = 4), including the only grade 3 AE observed in the highest dose group, do not seem to be drug-related but rather associated to the mode of application. Vasovagal events have been reported during uterine cervix manipulation with different techniques [21].

The local toxicity, as evidenced by the total number of these adverse events, was dose-dependent. Importantly, cervix examination 15 days after treatment showed that all these local AE had completely disappeared. Thus, the short-lasting of the local AE observed make this approach safe to treat cervical malignancies.

The standard treatments for HSIL patients, such as LEEP, laser, cold knife conization and cryotherapy yield satisfactory results. However, these procedures are not exempt of complications and undesired effects such as cervical obstruction, which is mostly linked to fertility limitations [22]. Events of this magnitude were not observed during the intralesional CIGB-300 administration and the genital area integrity was preserved. In fact, four pregnancies were achieved, two of them in previous apparently infertile women.

Products such as imiquimod, administered in a gel formulation has been extensively used to treat intraepithelial lesions and genital warts [23], but local adverse reactions leading to treatment interruption in some patients have been also reported [24]. In the present study, neither the local nor systemic CIGB 300-related toxicity lead to treatment interruption, at least with this therapeutic regimen. Importantly, long-term follow-up indicated that neither local nor systemic CIGB-300-related toxicity appeared in patients after 3, 6 and 12 months of completion the drug administration.

The promising antitumor effect of CIGB-300 found in preclinical studies, lead to the hypothesis that this pro-apoptotic peptide could exhibit anti-neoplastic effect on cervical malignancies when administered directly on the transformed cervix epithelium. The results obtained seem consistent with the initial hypothesis. For instance, the colposcopic end-point demonstrated a significant response rate in the entire CIGB-300 dose range as evidenced by the TLA reduction and the responder rates in all the cohorts after a short period of time (15 days). Further studies should determine whether longer treatment or follow-up periods are required for maximal efficacy. Similarly and equally important is that CIGB-300 treatment lead to a full histological regression in 19% of the patients. This means that CIGB-300 can be able to exhibit a beneficial effect in terms of the cure or reduction of the initial lesion. According to the natural history of the disease, spontaneous regression of NIC II and III after several months is found in 43% and 32% of the patients, respectively [25]. Contrarily, the histological and colposcopic regression rates observed in this study seem to be drug-related as they occurred in a very short period (15 days). Additionally, no recurrences appeared during one-year follow up. However, efficacy was not the main objective of this study. Therefore such results can just be taken as preliminary. It is additionally difficult to evaluate each case according to histology since the initial biopsy was taken from the apparently more representative zone of the lesion but the final biopsy comprises the whole lesion, so this last evaluation was more exhaustive. Future placebo-controlled studies are needed to asses this criterion since these lesions are usually heterogeneous and not treatment-related changes in histological degree in the same patient can occur.

HPV status has been considered as a robust and predictive biomarker of NIC recurrence and evolution to cancer in many clinical trials [26,27]. The result of this biomarker evaluation in this study showed that CIGB-300 exhibited an objective effect on virus presence. Forty-eight percent of initially HPV-positive patients experienced a complete virological response in the residual lesion after CIGB-300 administration. Although not all the cervix area was checked for HPV infection after drug administration, the fact that undetectable HPV levels were verified at the colposcopically directed biopsy is of great interest. The anti-HPV response observed in some patients could be reasonably explained by a potential apoptotic effect that eliminated the HPV-transformed cells during the consecutive 5-day CIGB-300 regimen.

Other molecular biomarkers (apoptosis mediators, CK2 phosphorylation status) were not studied because the second biopsy was taken 15 days after the last administration to coincide with the therapeutic cone. At that moment it is unlikely that these pharmacodynamic markers would have given any significant result since their changes are transiently regulated. An additional biopsy, close to end of treatment (day 5 or 6) would had been necessary for that purpose. However, this intervention was not ethically feasible and at the same time could have influence on the clinical outcome two weeks later.

This report describes the first clinical trial where a drug has been used to target the CK2 phosphorylation domain. The data reinforce the concept that CK2 phosphorylation represents a target with perspectives for cancer therapy, provide an early proof-of-principle of clinical benefit of a CK2 inhibitor to treat cervical malignancies, and suggest that CIGB-300 could represent a candidate drug for targeted cancer therapy.

## Conclusion

The CIGB-300 is a peptide-based drug that was safe and well tolerated when administered by intralesional injections. Neither a specific maximum-tolerated dose nor dose-limiting toxicity was identified, but given the efficacy signs observed at all dose levels tested, the total dose range from 14 to 490 mg is recommended for phase 2 trials. CIGB-300 treatment indicated clinical activity, as evidenced by the colposcopy, histology and the effective anti-HPV response. Such clinical effects should be confirmed in further controlled trials.

## Abbreviations

AE: adverse event; CIN: Cervical Intraepithelial Neoplasia; CK2: Casein kinase 2; DLT: Dose limiting toxicity; DNA: deoxyribonucleic acid; FIGO: Fédération Internationale de Gynécologie et Obstétrique; HPV: Human Papillomavirus; HSIL: High grade squamous intraepithelial lesion; LEEP: Loop electrosurgical excision procedure; MIC: Microinvasive carcinoma); MTD: Maximum Tolerated Dose; PCR: polymerase chain reaction; QR: interquartile range; RECIST: Response Evaluation Criteria in Solid Tumors; SD: standard deviation; TLA: total lesion area.

## Competing interests

Authors IB, CV, TR, and PLS are employees of the Center for Biological Research, which is part of the Center for Genetic Engineering and Biotechnology (CIGB), Havana, where CIGB-300 is produced. LH, SEP, and BEA are employees of CIGB itself. RG, DFA, and HS are employed by Biorec. SEP is the main author of the patent WO 03/054002; US patent # 7374767 that sustain this project. The rest of the authors have no conflict of interest.

## Authors' contributions

AMS and AS were the main clinical investigators. ID participated in the study design, coordination and monitoring, and in manuscript writing. CG and AD took care of patient management and adverse event evaluation. CV participated in the study design, statistics, and results analyses. TR carried out HPV studies. DC did the cytological and histological evaluations. PLS took part in the design, results analyses and manuscript writing. RG, DFA, LH, HS, SEP, and BEA took part in the design and analyses of results. All authors read and approved the final manuscript.

## Pre-publication history

The pre-publication history for this paper can be accessed here:

http://www.biomedcentral.com/1471-2407/9/146/prepub
